# Revision of the Pharmacopoeia

**Published:** 1899-07

**Authors:** H. C. Wood

**Affiliations:** President of the National Convention for Revising the U. S.


### Abstracts and Selections.

### Revision of the Pharmacopoeia.

To All Whom it May Concern:—In accordance with the instructions given by the resolutions passed at the meeting of the National Convention for Revision of the Pharmacopœia of the United States of America, held in Washington, A. D. 1890, I herewith give notice that a general convention for the revision of the pharmacopœia of the United States of America will be held in the city of

Washington, D. C., beginning on the first Wednesday in May, 1900. It is requested that the several bodies represented in the convention of 1880 and 1890, and also such other incorporated State medical and pharmaceutical associations, and incorporated colleges of medicine and pharmacy, as shall have been in continuous operation for at least five years immediately preceding this notice, shall each elect delegates, not exceeding three in number; and that the surgeon-general of the army, the surgeon-general of the navy and the surgeon-general of the Marine Hospital Service, shall appoint, each, not exceeding three medical officers to attend the aforesaid convention.

It is desired that the several medical and pharmaceutical bodies, and the medical departments of the army, navy and Marine Hospital Service shall transmit to me the names and residences of their respective delegates so soon as said delegates shall have been appointed, so that a list of the delegates to the convention may be published in accordance with the resolutions passed at the 1890 convention for the revision of the pharmacopœia, in the newspapers and medical journals in the month of March, 1900.

Finally, it is further requested that the several medical and pharmaceutical bodies concerned, as well as the medical departments of the army, navy and Marine Hospital Service, shall submit the present pharmacopœia to a careful revision, and that their delegates shall transmit the result of their labors to Dr. Frederick A. Castle, 51 West 58th Street, New York City, Secretary of the Committee of Revision and Publication of the United States Pharmacopœia, at least three months before May 2, 1900, the date fixed for the meeting of the convention.

H. C. Wood, M. D.,

President of the National Convention for Revising the U. S. Pharmacopœia, held in Washington, D. C., A. D. 1890.

University of Pennsylvania, Philadelphia, Pa., May 1, 1899.

---

**The Journal** acknowledges with pleasure the receipt from Messrs. Arthur Peter & Co., the big Louisville drug house, a beautiful souvenir in the shape of a "medical case"—they call it. It is a pressed morocco leather folder, with pockets for cards, or powders, and compartments for stamps. It is first-class, and "good to have in the house,"—as are also their well known and popular pharmacal preparations,—*Syrup Roborans* and *Peter's Peptic Essence Compound.*

